# Overexpression of *TaPSKR1L-6A* improves resistance to sharp eyespot and increases lignin accumulation in wheat

**DOI:** 10.3389/fpls.2025.1653282

**Published:** 2025-10-17

**Authors:** Haijun Qi, Yuran Hao, Xiuliang Zhu, Zengyan Zhang

**Affiliations:** ^1^ Institute of Special Animal and Plant Sciences, Chinese Academy of Agricultural Sciences, Changchun, China; ^2^ Nanfan Research Institute, CAAS, Sanya, Hainan, China; ^3^ The National Key Facility for Crop Gene Resources and Genetic Improvement, Institute of Crop Sciences, Chinese Academy of Agricultural Sciences, Beijing, China; ^4^ Northwest Agricultural and Forestry University, Yangling, China

**Keywords:** common wheat (Triticum aestivum), lignin synthesis, phytosulfokine receptor 1-like, resistance, sharp eyespot

## Abstract

The soil-borne fungi *Rhizoctonia cerealis* is one of the major pathogens for the economically-important diseases sharp eyespot of common wheat (*Triticum aestivum*). Certain *phytosulfokine receptor 1* (*PSKR1*) genes mediate resistance to diseases caused by biotrophic/hemibiotrophic pathogens in several plant species. Yet, none of wheat *PSKR1* genes with positive effect on the innate immune responses to *R. cerealis* has been reported. Here, we characterize the phytosulfokine receptor 1-like gene TaPSKR1L-6A on wheat chromosome 6A, and determined functional role of it in wheat defense against *R. cerealis*. We used RNA-seq, BSMV-induced gene silencing and transgenic wheat to investigate the role of TaPSKR1L-6A in resistance to *R. cerealis*. The results showed that TaPSKR1L-6A has a resistance effect on *R. cerealis*. Transcription analysis showed that its expression level was upregulated when infected with sheath blight. Overexpression of TaPSKR1L-6A significantly increased sheath blight resistance, upregulated the expression of lignin synthesis and metabolism pathway genes, and increased lignin accumulation, which was opposite to the results of silencing TaPSKR1L-6A. Collectively, these results clearly suggest that TaPSKR1L-6A mediates resistance to sharp eyespot by activating the expression of several lignin synthesis-related genes and increasing lignin accumulation. Thus, *TaPSKR1L-6A* is a promising gene for improving wheat resistance against sharp eyespot. This study sheds light on wheat defense mechanisms against *R. cerealis*.

## Introduction

Bread wheat (*Triticum aestivum* L.) is a globally vital staple crop, supplying approximately one-fifth of human dietary calories (International Wheat Genome Sequencing Consortium, 2018). Sharp eyespot, a serious disease primarily caused by the soil-borne fungus *Rhizoctonia cerealis*, affects grain cereals including bread wheat in major wheat-growing regions such as Australia, the USA, and China (Hamada et al., 2011). Therefore, the control of sharp eyespot is extremely urgent. Utilizing resistant wheat varieties represents a long-established, environmentally friendly, and economical control strategy. However, the majority of popular cultivars susceptible to this disease in China (Wu et al., 2017; Li et al., 2022). Therefore, identifying resistance genes and elucidating the underlying molecular mechanisms are vital for efficiently breeding wheat varieties with resistance to sharp eyespot.

Past studies indicate that resistance to sharp eyespot in wheat is controlled by multiple resistant genes (Wu et al., 2022). Several genes involved in the resistance response to sharp eyespot have been identified in wheat through approaches such as transcript expression analysis, gene silencing, and overexpression. For example, studies by Zhang et al. (2007) and Zhu et al. (2014) demonstrated that the pathogen-induced wheat ERF transcription factors TaERF3 and TaPIE1 transmit ethylene signals, upregulate the expression of defense genes, and consequently enhance wheat resistance to sharp eyespot. Shan et al (2016) and Liu et al. (2020) discovered, through experiments including Virus-Induced Gene Silencing (VIGS) and overexpression, that the *R. cerealis*-induced MYB transcription factor gene *TaRIM1* and the GATA transcription factor gene *TaGATA1* positively regulate wheat resistance to sharp eyespot. This regulation is associated with their ability to significantly upregulate the transcription levels of defense genes. Zhu et al. (2017) functionally analyzed a wheat *R. cerealis*-induced Nucleotide-Binding Site Leucine-Rich Repeat (NBS-LRR) class Resistance (R) gene, *TaRCR1*, using VIGS and transgenic wheat. They found that this gene enhances wheat resistance to the pathogen by promoting reactive oxygen species (ROS) scavenging and the expression of defense genes. Wang et al. (2020a), employing gene silencing and heterologous overexpression in *Arabidopsis thaliana*, revealed that TaELP4 increases the expression of defense genes and wheat plant resistance to sharp eyespot by regulating the acetylation of defense gene proteins. Wang et al. (2021) characterized the function of TaMKK5 in wheat resistance to sharp eyespot using overexpression and knockdown methods. They proposed that the TaMKK5-TaMPK3-TaERF3 signaling cascade regulates this resistance. Research by Guo et al (2021) indicated that the membrane protein TaCRK3 can directly inhibit the growth of *R. cerealis* through its extracellular DUF26 domain and activate the expression of disease resistance-related genes via its intracellular kinase domain. VIGS analysis by Qi et al. (2021a, 2021b) showed that silencing *TaWAK7D* and *TaWAK-6D* suppresses the expression of downstream defense response genes and attenuates wheat resistance to sharp eyespot. Zhu et al. (2022) found that the transcript abundance of the glycosyltransferase-encoding gene *TaSTT3b-2B* is positively correlated with wheat resistance to sharp eyespot. In summary, several candidate genes positively regulating wheat resistance to sharp eyespot have currently been identified. However, the resistance network of wheat against sharp eyespot remains to be fully elucidated.

To defend against pathogens, plants evolved an array of receptor-like kinases (RLKs) or receptor-like proteins to perceive pattern-associated molecular patterns (PAMPs) or damage-associated molecular patterns (Boutrot and Zipfel, 2017; Rhodes et al., 2021; Wang et al., 2020b; Zipfel, 2014). The disulfated pentapeptide Phytosulfokine (PSK) is an important signaling molecule, and can act as a PAMP in plant defenses to pathogen infection (Sauter, 2015; Yang et al., 2019). It is perceived by the LRR RLKs PSKR1 (phytosulfokine receptor 1) and PSKR2 (phytosulfokine receptor 2) (Matsubayashi et al., 2002; Zhang et al., 2018). PSK has been shown to bind to the island domain of PSKR1 that intersects between LRR17 and LRR18 of the extracellular receptor domain, from where the signal is transmitted to the intracelluar receptor kinase domain (Matsubayashi et al., 2002; Wang et al., 2015; Kaufmann et al., 2017). PSK binding enhances PSKR heterodimerization with somatic embryogenesis receptor-like kinases (SERKs) (Wang et al., 2015; Hohmann et al., 2018). Recently, *PSKR1s* in *Arabidopsis*, tomato and rice have been implicated in plant innate immune responses to pathogens. In *Arabidopsis*, *AtPSKR1* could enhance defense against the necrotrophic fungal pathogen *Alternaria brassicicola* (Mosher et al., 2013). In tomato, *SlPSKR1* participated in the auxin-mediated pathways and positively contributed to innate immunity to infection of the necrotrophic fungus *Botrytis cinerea* (Zhang et al., 2018). In rice, *OsPSKR1* was positively involved in resistance to *Xanthomonas oryzae pv. oryzicola* by regulating the expression of pathogenesis-related genes in salicylic acid pathway (Yang et al., 2019). Our laboratory showed that *TaRLK‐6A*, a *PSKR1-*like gene locating on the chromosome 6A, could interact with *TaSERK1* positively modulate the resistance to *Fusarium* crown rot in wheat (Qi et al., 2024). However, the functions of PSKR1 involved in resistance to *R. cerealis*, and lignin accumulation in wheat were scarcely reported.

In this study, by means of RNA-sequencing (RNA-Seq) of wheat against *R. cerealis*, barley stripe mosaic virus-induced VIGS, as well as transgenic assays, we found that the expression of *TaPSKRL1-6A* was significantly induced after *R. cerealis* infection and the induction was higher in sharp eyespot-resistant wheat cultivars than in susceptible cultivars. Overexpression of *TaPSKRL1-6A* significantly enhanced resistance to sharp eyespot, increasing the expression lignin synthesis genes, and lignin content in transgenic wheat, while silencing of *TaPSKRL1-6A* displayed the opposite effects. These findings not only demonstrate positive contribution of *TaPSKRL1-6A* to sharp eyespot resistance, but also provide insights on functional roles of PSKR1-like in innate immunity of plant species, especially wheat.

## Results

### TaPSKR1L-6A was involved in the wheat defense response to *R. cerealis*

**Figure 1:**
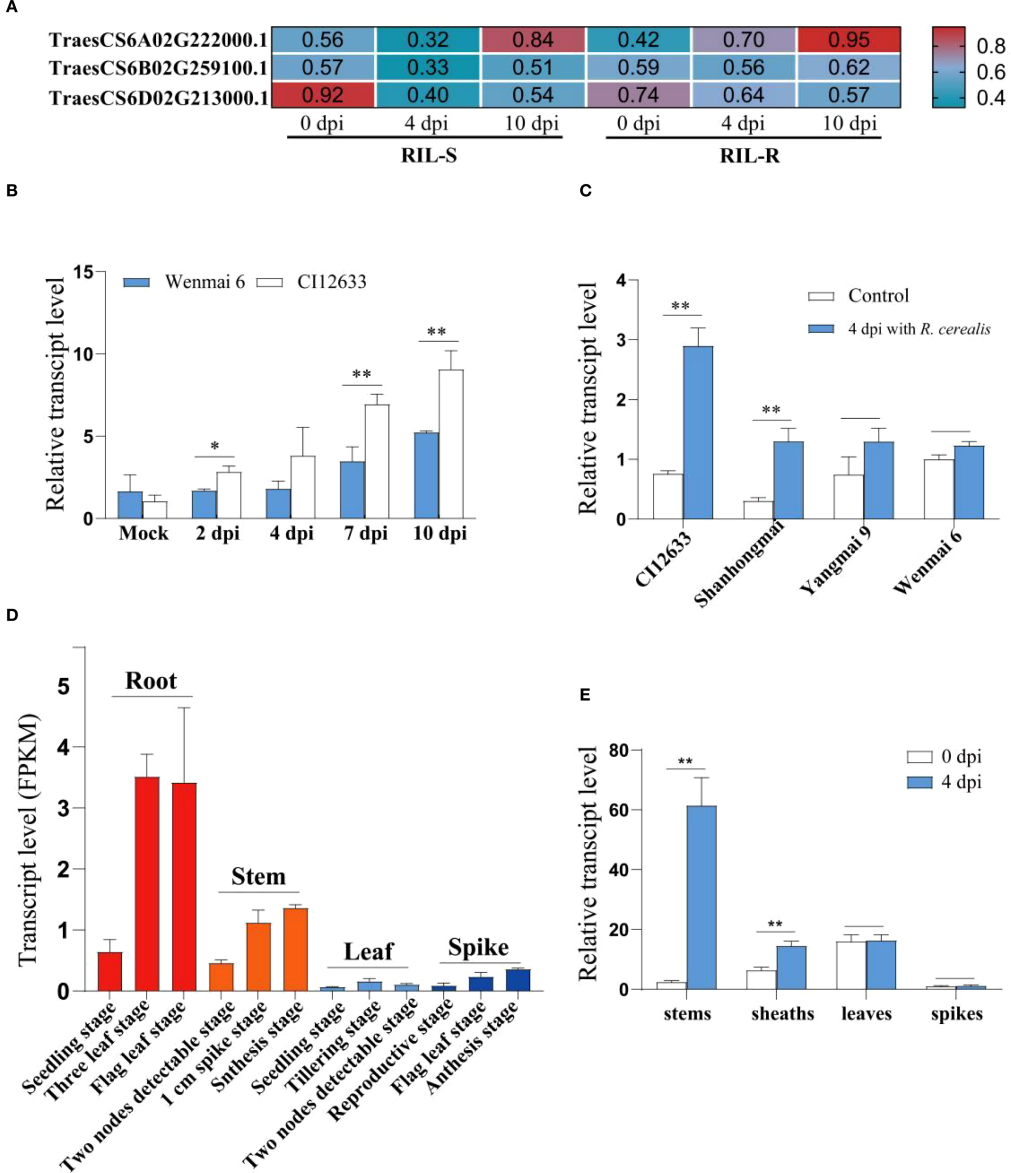
The expression pattern of *TaPSKR1L-6A.* **(A)** The transcript change of *TaPSKR1L-6A* in the recombinant inbred lines (RILs) upon *R. cerealis* infection. **(B)** Transcript patterns of *TaPSKR1L-6A* with *R. cerealis* infection. The *TaPSKR1L-6A* transcript level of wheat cultivar Wenmai 6 at non-treatment was set to 1. **(C)** Expression patterns of *TaPSKR1L-6A* in 4 wheat cultivars after *R. cerealis* infection. The expression level of *TaPSKR1L-6A* in Wenmai 6 at 0 dpi was set to 1. *TaActin* was used as an internal control gene (t-test: *P< 0.05; **P< 0.01). Bars indicate SEs of the mean. **(D)** Tissue-specific expression patterns of *TaPSKR1L-6A*, *TaPSKR1L-6B*, and *TaPSKR1L-6D* in the wheat cultivar Chinese Spring as determined from RNA-seq online data (http://www.wheat-expression.com, International Wheat Genome Sequencing Consortium (IWGSC), 2014). **(E)** Transcript pattern of *TaPSKR1L-6A* in stems, sheathes, leaves and spikes of CI12633 at 4 dpi with *R. cerealis* of non-treatment. *TaActin* was used as an internal control gene (t-test: *P< 0.05; **P< 0.01).

By mining the RNA-seq data from resistant and susceptible lines of recombinant inbred lines (RILs) derived from the cross Shanhongmai × Wenmai 6, the gene with ID: TraesCS6A02G222000.1, named as *TaPSKR1L-6A*, was identified to be up-regulated in the resistant RILs relative to the susceptible RILs after *R. cerealis* strain Rc207 inoculation. The gene transcript level was higher by 2.18- and 1.14-fold in the resistant RILs relative to the susceptible lines at 4- and 10-days post inoculation (dpi) with the fungus, respectively (**Figure 1A**). RT-qPCR analysis showed that after inoculation with *R. cerealis*, *TaPSKR1L-6A* transcript level was significantly greater in resistant wheat cv.CI12633 than the susceptible wheat cultivar Wenmai 6 (**Figure 1B**), implying that the gene may be involved in the early defense response to the fungal infection. The analyses were in agreement with the gene transcript trend in the RNA-seq data. Importantly, among the four different wheat cultivars, the *TaPSKR1L-6A* transcript level was the highest in *R. cerealis* resistant wheat cultivar CI12633, followed by the resistant wheat Shanhongmai, but was the lowest in two highly susceptible cultivars Yangmai 9 and Wenmai 6 (**Figure 1C**). This result suggested that the transcript abundance of *TaPSKR1L-6A* was correlated to the resistant degree of the four wheat cultivars.

By querying the tissue-specific expression database of Chinese Spring wheat, it was found that the expression level of this gene is relatively high in root and stem tissues, but lower in leaves and spikes (**Figure 1D**). This expression pattern is consistent with the infection and occurrence sites of sharp eyespot. And the expression patterns of different tissues showed that at 4 dpi with *R. cerealis*, the higher-level change appeared at the stems and sheaths of CI12633 seedlings (**Figure 1E**), where sharp eyespot disease usually occurs. These data suggested that *TaPSKR1L-6A* might participate in wheat resistance responses to *R. cerealis* infection.

Sequencing of *TaPSKR1L-6A* promoter in 61 wheat cultivars indicated that a SNP (T to C) at -1191 sit formed two *TaPSKR1L-6A* haplotypes in this population (**Supplementary Figure S1A**). To compare the contributions of *TaPSKR1L-6A Hap I* and *Hap II* to wheat sharp eyespot resistance, we examined the resistance of the population to *R. cerealis*. As shown in **Supplementary Figure S1B**, the disease index of wheat cultivars containing *Hap I* was significantly lower than that of cultivars containing *Hap II*. These results suggest that the *TaPSKR1L-6A Hap I* allelic variant is associated with higher sharp eyespot resistance compared with *TaPSKR1L-6A Hap II*. Curiously, only 9.84% of wheat cultivars had *Hap I* (**Supplementary Figure S1C**), suggested that this haplotype was not fully selected during the modern wheat breeding process.

### TaPSKR1L-6A positively regulates resistance to sharp eyespot in wheat

**Figure 2:**
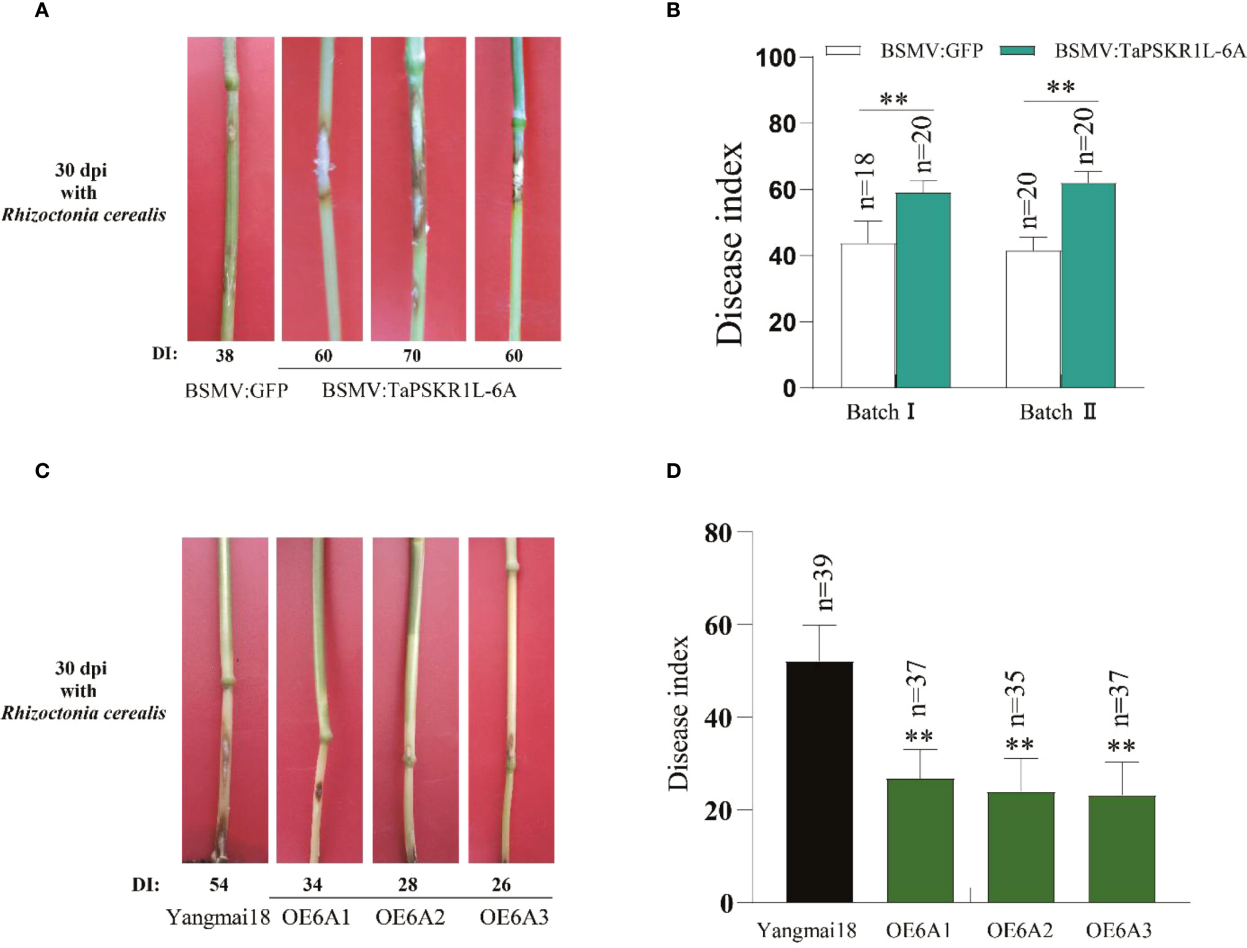
TaPSKR1L-6A positively regulates resistance to sharp eyespot in wheat. **(A)** sharp eyespot symptoms on *TaPSKR1L-6A*-silenced and BSMV: GFP-infected (control) CI12633 plants at 30 dpi with *R. cerealis*. **(B)** Disease index of *TaPSKR1L-6A*-silenced or control CI12633 plants at 30 dpi with *R. cerealis* in two independent batches (t-test: **P< 0.01). Bars indicate SEs of the mean. **(C)** Typical symptoms of sharp eyespot in the three *TaPSKR1L-6A* overexpressing and WT ‘Yangmai18’ lines at 30 dpi with *R. cerealis*. **(D)** Disease indexes of *TaPSKR1L-6A* overexpressing and WT ‘Yangmai18’ lines. Bars indicate SEs of the means (n varies for each column and is shown in each case directly on the graphs), and asterisks indicate significant differences between WT and transgenic lines using Student’s t-tests (**P< 0.01).

To investigate *TaPSKR1L-6A*-mediated resistance to sharp eyespot in wheat, we generated *TaPSKR1L-6A*-silenced plants by Barley stripe mosaic virus (BSMV) mediated silencing assay (VIGS). The *TaPSKR1L-6A-*silenced and BSMV: GFP-infected(control)wheat plants were individually inoculated with *R. cerealis* strain RC207. At 30 dpi with *R. cerealis*, compared with BSMV: GFP-infected CI12633 plants, the stems of *TaPSKR1L-6A* ***-*** silenced CI12633 plants exhibited more serious disease severity of sharp eyespot with larger necrotic areas (**Figure 2A**). In the disease tests of two VIGS batches, the disease indices of *TaPSKR1L-6A*-silenced CI12633 plants were 59.2 and 62, respectively, while that of BSMV: GFP-infected CI12633 plants were 43.8 and 41.6, respectively (**Figure 2B**). These data suggested that the expression of *TaPSKR1L-6A* is required for resistance of wheat to *R. cerealis.*

To further examine the function of *TaPSKR1L-6A* in wheat resistant response to sharp eyespot, we generated *TaPSKR1L-6A* transgenic plants. These plants in T_2_ generation were further inoculated with *R. cerealis* strain Rc207 to assess the resistance role of *TaPSKR1L-6A*. At 30 dpi, the sharp eyespot lesion sizes were smaller on the stems of *TaPSKR1L-6A* overexpressing wheat plants than the WT plants (**Figure 2C**). In disease assessments, the disease indexes of three *TaPSKR1L-6A-6* overexpression wheat lines were 26.85, 23.93, and 23.25, respectively; whereas the disease index of the WT recipient ‘Yangmai18’ plants was 52.16 (**Figure 2D**; **Supplementary Table S1**). These results indicated that compared to WT plants, overexpression of *TaPSKR1L-6A* significantly increased resistance to sharp eyespot in the transgenic wheat lines.

### TaPSKR1L-6A positively regulate lignin synthesis in wheat

**Figure 3:**
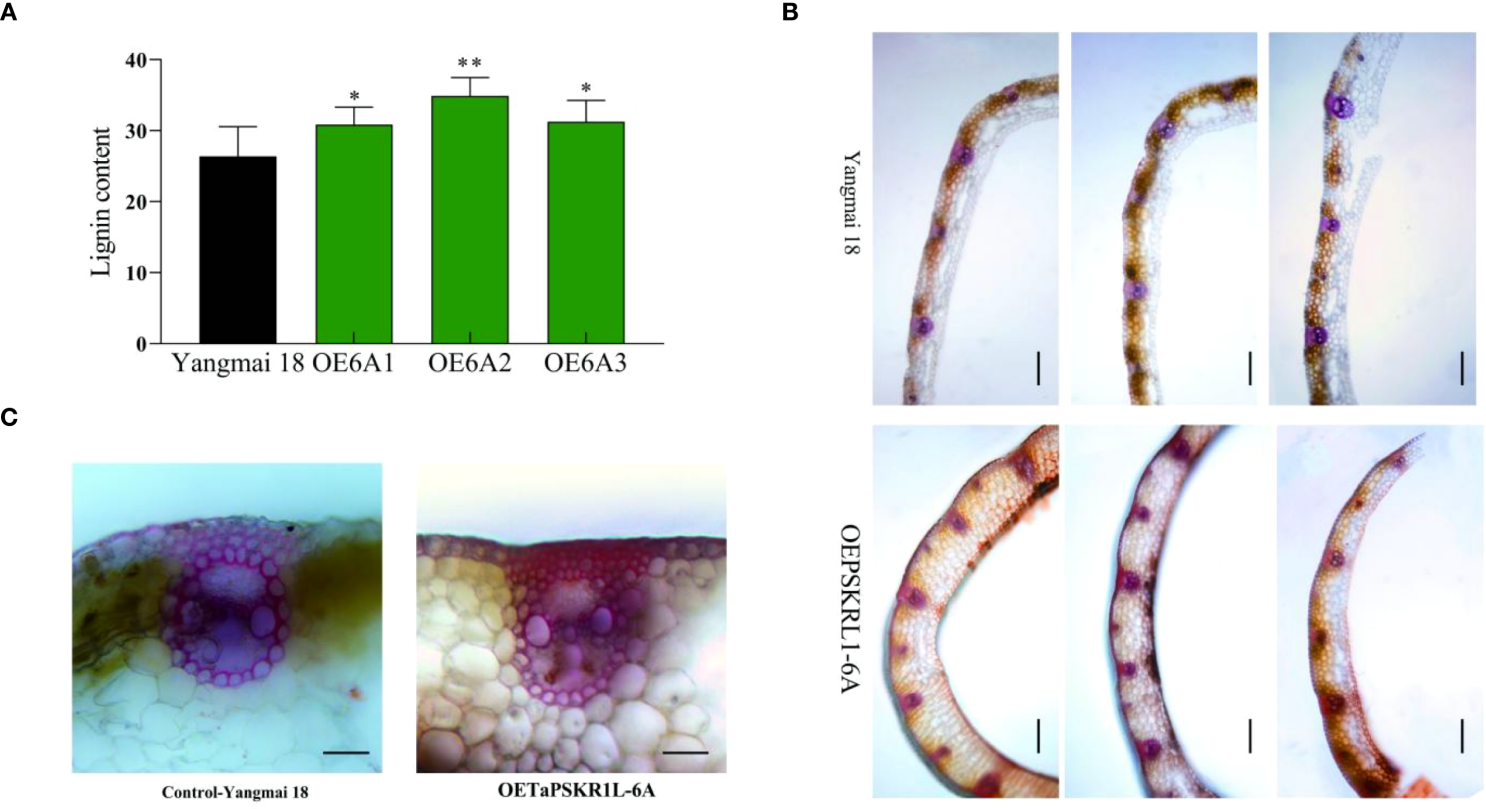
*TaPSKR1L-6A* overexpression increases lignin content in transgenic wheat plants. **(A)** Lignin content was higher in *TaPSKR1L-6A* overexpression plants than WT ‘Yangmai 18’ by quantitative measurement. Lignin content was quantitatively measured by using the lignin ELISA Kit (ChemFaces, China) according to the manufacturer’s protocol. Three biological replicates per line were averaged (t-test; **P< 0.01,*p<0.05). Bars indicate SEs of the means. **(B)** Lignin staining of the *TaPSKR1L-6A* overexpression and WT ‘Yangmai 18’ plants. Hand-cut sections from leaf sheaths were subjected to Mäule staining. Bar=100 μm. **(C)** The results of Wiesner staining showed that *TaPSKR1L-6A* overexpressing wheat plants had more sclerenchyma cells than WT ‘Yangmai 18’ plants. Bar=50 μm.

During the evaluation on resistance of transgenic wheat to sharp eyespot, we found the stem strength in *TaPSKR1L-6A* overexpression wheat plants seemed to be higher than that in WT ‘Yangmai18’ plants. Therefore, we analyzed the lignin content of *TaPSKR1L-6A* overexpression and WT wheat plants. The results showed that the lignin content in the *TaPSKR1L-6A* overexpression transgenic plants was significantly higher than that in the WT plants (**Figure 3A**). Furthermore, hand-cut sections from leaf sheath of the *TaPSKR1L-6A* overexpression and WT plants were subjected to Wiesner and Mäule staining. As a result, compared to the WT samples, the samples of the *TaPSKR1L-6A* overexpression plants displayed more strengthened staining (red-brown) (**Figure 3B**), which was indicative of a higher lignin level. Additionally, compared with the WT plants, the *TaPSKR1L-6A* over-expression wheat plants had more sclerenchyma cells, which also can be detected by Mäule and Wiesner (**Figures 3B, C**) staining. These results indicated that overexpression of *TaPSKR1L-6A* enhanced lignin accumulation. Eventually, we measured the stem mechanical (breaking) strength of the second basal internode in *TaPSKR1L-6A* overexpressing and ‘Yangmai18’ wheat plants at harvest stage. The results showed that, *TaPSKR1L-6A* overexpressing lines exhibited significantly increased mechanical strength than those in ‘Yangmai18’ wheat plants (**Supplementary Figure S2**).

**Figure 4:**
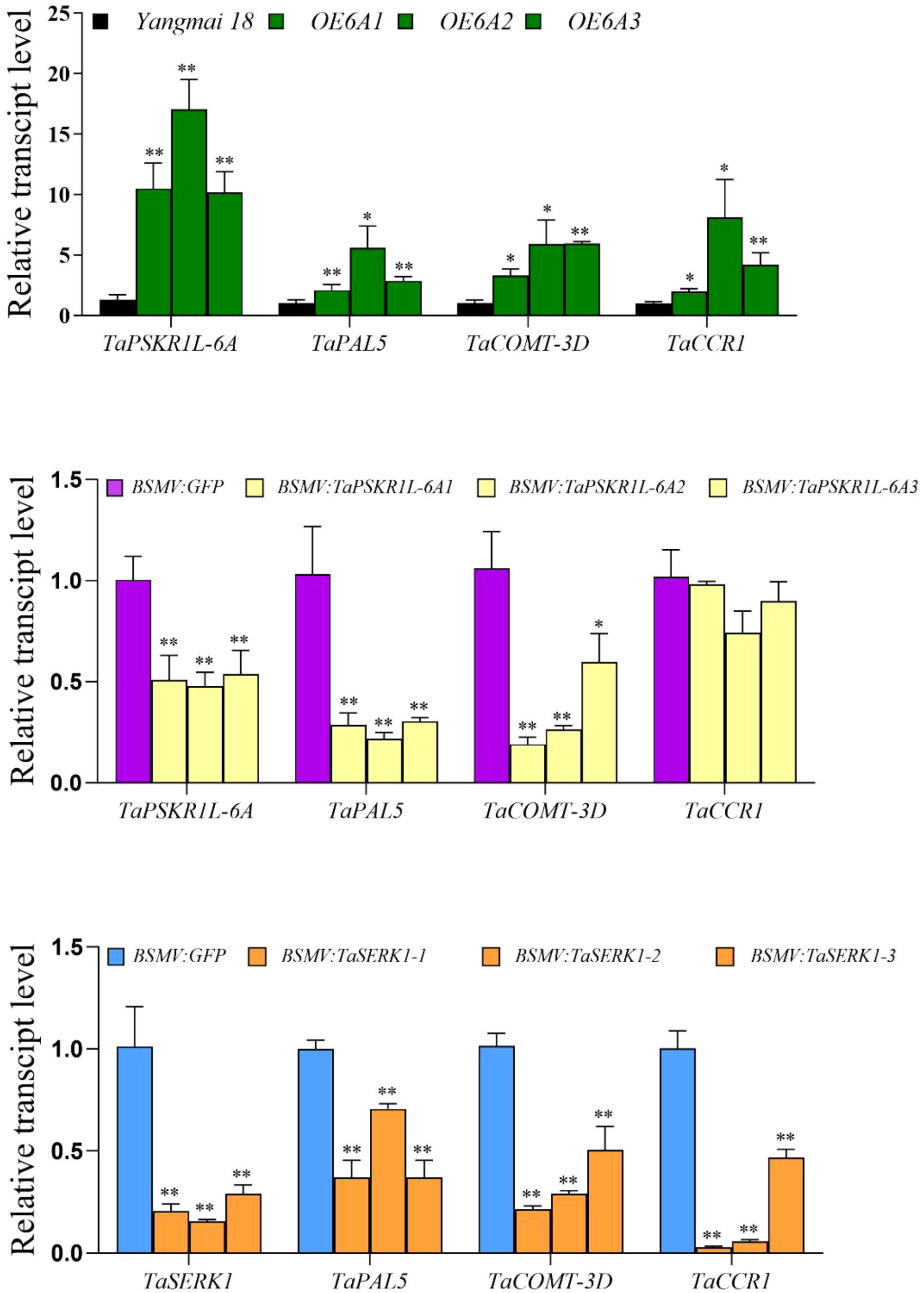
Lignin synthesis genes were regulated by *TaPSKR1L-6A* and *TaSERK1*. **(A)** Relative transcript abundances of three lignin synthesis genes in *TaPSKR1L-6A* over-expressing wheat plants and WT wheat ‘Yangmai 18’ plants. **(B)** Relative transcript abundances of three lignin synthesis genes in BSMV: GFP -infected and BSMV: *TaPSKR1L-6A*-silenced wheat CI12633 plants. **(C)** Relative transcript abundances of three lignin synthesis genes in BSMV: GFP-infected and BSMV: *TaSERK1*-silenced wheat plants at 4 dpi with R. cerealis. All samples were taken from sheaths of wheat plants. Statistically significant differences were determined based on three replications using a t-test (*P< 0.05; **P< 0.01). Bars indicate the SEs of the mean. *TaActin* was used as the internal reference gene to normalize the relative expression.

To investigate why the lignin content is higher in *TaPSKR1L-6A* overexpression plants, we measured the expression of several lignin synthesis-related genes in *TaPSKR1L-6A-* overexpression and silenced wheat plants as well as their controls. The results showed that transcript levels of lignin synthesis-related genes, *TaPAL5*, *TaCOMT-3D*, and *TaCCR1*, were higher in *TaPSKR1L-6A* overexpression plants than in the WT (control) plants (**Figure 4A**). While transcript levels of *TaPAL5* and *TaCOMT-3D* were significantly lower in *TaPSKR1L-6A*-silenced plants than in control BSMV: GFP-infected plants, but transcript levels of *TaCCR1* had no significant difference between *TaPSKR1L-6A-*silenced plants and control plants (**Figure 4B**). The results indicated *TaPSKR1L-6A* positively regulated lignin synthesis through modulating the expression of at least *TaPAL5* and *TaCOMT-3D.*

Our previous article demonstrated that TaPSKR1L-6A could interact with TaSERK1 to co-regulate wheat resistance to *Fusarium* crown rot (Qi et al., 2024). To analysis whether the TaSERK1 was related with lignin synthesis in wheat. We generated *TaSERK1*-silenced wheat plants and tested transcripts of the three lignin synthesis-related genes (*TaPAL5*, *TaCOMT-3D*, and *TaCCR1*) that are up-regulated by *TaPSKR1L-6A*. The results showed that transcript levels of *TaPAL5*, *TaCOMT-3D*, and *TaCCR1* were significantly lower in *TaSERK1*-silenced plants compared with the control plants (**Figure 4C**). These data suggest that *TaPSKR1L-6A* and *TaSERK1* co-regulated the lignin synthesis to enhance stem strength.

## Discussion

Recently, sharp eyespot, mainly caused by soil-borne fungus *R. cerealis*, has become an important devastating disease of wheat in many regions of the world. Given the polygenic nature of sharp eyespot resistance, research should focus on expanding the wheat disease resistance network, identifying major QTL, and pyramiding multiple resistance genes using molecular markers to gradually enhance wheat resistance to this disease. Although the PSKR1 genes have been reported to involve in innate immunity against pathogens in *Arabidopsis*, tomato, and rice (Mosher et al., 2013; Zhang et al., 2018; Yang et al., 2019b), the function role of *PSKR*1 in wheat resistance responses has not been reported yet. In this study, we identified *TaPSKR1L-6A* as a candidate resistance gene of wheat against sharp eyespot, its transcript induction by infection of *R. cerealis* was higher in the tested sharp eyespot-resistant wheat cultivars than those in the susceptible wheat cultivars. Further functional dissection results showed that overexpression of *TaPSKR1L-6A* significantly enhanced wheat resistance to sharp eyespot, while silencing of *TaPSKR1L-6A* compromised resistance of wheat to sharp eyespot. Taken together, these results indicated that *TaPSKR1L-6A* confers wheat resistance to sharp eyespot and thus *TaPSKR1L-6A* is a promising gene for improving wheat resistance to sharp eyespot.

Downstream pathway of the PSKR-triggered immunity is not well understood (Yang et al., 2019). In *Arabidopsis thaliana*, *AtCCR1* is important for lignin synthesis (Meester et al., 2018). In rice, *OsPALs* were reported to mediate resistance to brown plant hopper by regulating the biosynthesis and accumulation of salicylic acid and lignin (He et al., 2020). In wheat, overexpression of *TaCOMT-3D* was demonstrated to enhance wheat resistance against sharp eyespot and increase stem mechanical strength through promoting lignin accumulation, while silencing of *TaCOMT-3D* had opposite effects (Wang et al., 2018). In this study, the experiments indicated that overexpression of *TaPSKR1L-6A* elevated the expression of lignin synthesis genes (*TaPAL5*, *TaCOMT-3D*, and *TaCCR1*), increased lignin content/accumulation and the stem strength in the resistant transgenic wheat lines, while silencing of *TaPSKR1L-6A* had opposite effects. Moreover, the *TaPSKR1L-6A* overexpression plants had more sclerenchyma cells and deeper lignin staining compared to the WT plants, suggesting that overexpression of *TaPSKR1L-6A* enhanced lignin accumulation and promoted the development of vascular bundles.

**Figure 5:**
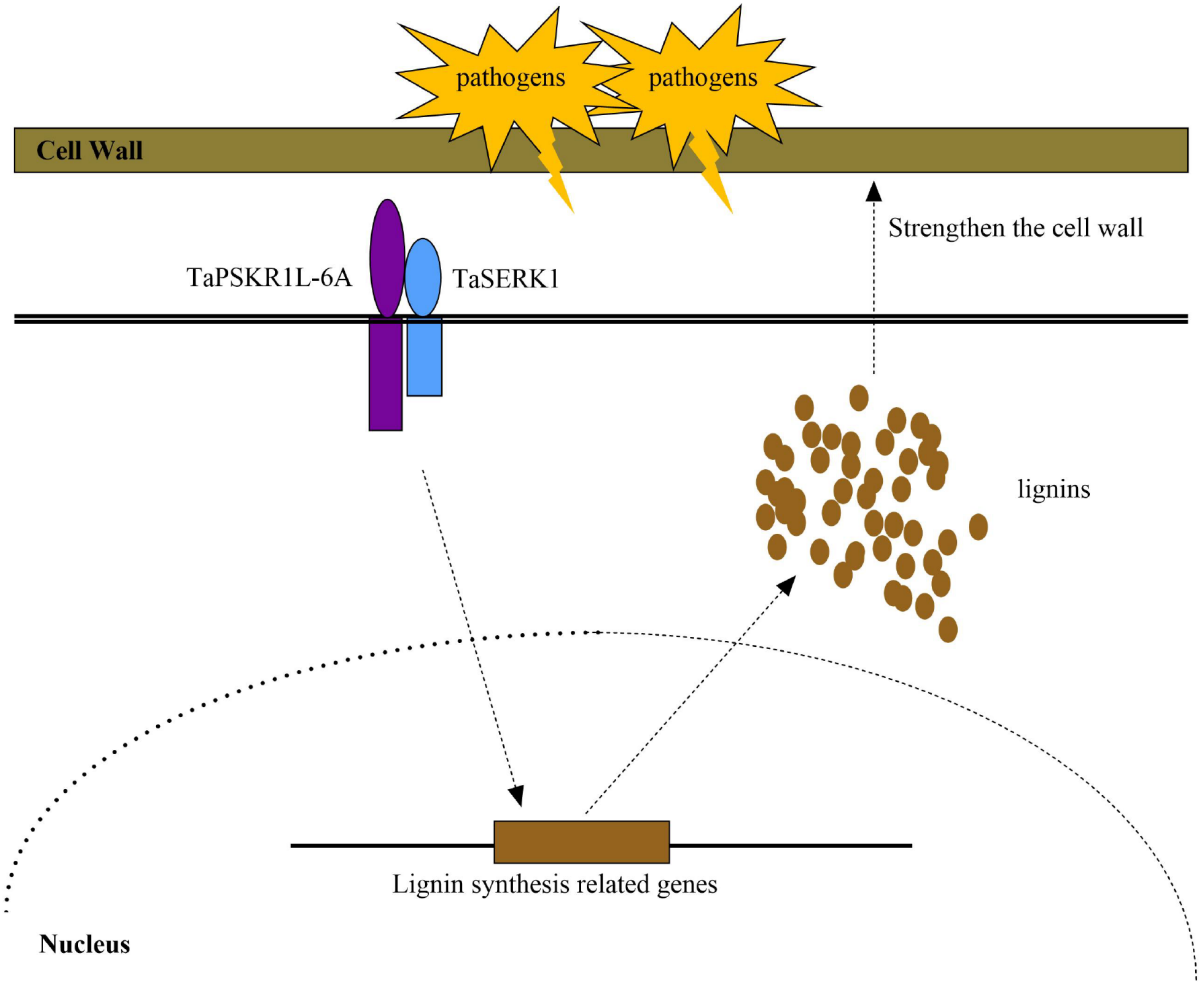
A working model of TaPSKR1L-6A improves resistance to sharp eyespot and increases lignin accumulation in wheat.

Our previous article demonstrated that TaPSKR1L-6A could interact with TaSERK1 to co-regulate wheat resistance to *Fusarium* crown rot (Qi et al., 2024). In this study, we found the expression levels of *TaPAL5*, *TaCOMT-3D*, and *TaCCR1* in *TaSERK1*-silenced plants were reduced compared to the control wheat plants. As the SERK proteins were reported to be related to the development of vascular bundles (Zhang et al., 2016; Li et al., 2019). Taken together, these results indicated that TaPSKR1L-6A, which interacted with TaSERK1, co-regulated lignin synthesis and stem strength, leading to enhanced resistance of wheat to sharp eyespot (**Figure 5**).

To our knowledge, this is the first investigation of PSKR1 or PSKR1L involvement in lignin synthesis and accumulation. The findings enrich the PSKR1/L signal pathway. Taken together, the current results enrich insights onto the PSKR-transducing signal pathways.

## Conclusions

We demonstrated that *TaPSKR1L-6A* could confer sharp eyespot resistance through activating the expression of lignin synthesis-related genes and increasing lignin accumulation in wheat. Overexpression of *TaPSKR1L-6A* significantly enhanced wheat resistance against sharp eyespot and promoting lignin synthesis/accumulation. By contrast, silencing of *TaPSKR1L-6A* and *TaSERK1* resulted in the opposite effects. To our knowledge, this is the first report about regulatory role of PSKR1/PSKR1L in lignin synthesis, and overexpression of a resistance gene improving wheat resistance to sharp eyespot. Thus, *TaPSKR1L-6A* was a promising gene for breeding wheat varieties with resistance to sharp eyespot. This study sheds light on wheat defense mechanisms against *R. cerealis*, and provides insights into functional roles of the PSKR1/PSKR1L in plant innate immunity, especially in the important crop wheat.

## Materials and methods

### Plant and fungal materials and treatments

Four wheat cultivars, including CI12633, Shanhongmai, Yangmai 9, and Wenmai 6, which exhibit different levels of resistance and susceptibility to sharp eyespot, were used to investigate *TaPSKR1L-6A* transcript profiles against *R. cerealis*. A middle resistant wheat cv. CI12633 was used for VIGS experiments. A middle susceptible wheat Yangmai 30 was used as the recipient material for genetic transformation experiments. All wheat seedlings were grown in a greenhouse at 23°C for 14 h in light, and 15°C for 10 h in darkness.

*Rhizoctonia cerealis* strain RC207, which is highly virulent in north China, was isolated by Prof. Jinfen Yu and Dr. Li Zhang (Shandong Agricultural University, China). The fungal strain was cultured on a PDA medium at 25°C in dark conditions. The sequences of all primers in this study are listed in **Supplementary Table S2**.

### RNA-Seq and RT-qPCR analysis

In this study, the RILs derived from the cross Shanhongmai × Wenmai 6, were used for RNA-Seq (RNA-sequencing) analysis as described by Guo et al. (2021). Primers of target genes for RT-qPCR were designed by Primer Premier 5 software and are listed in **Supplementary Table S2**. The RT-qPCR was performed using SYBR Green SuperReal PreMix (TIANGEN, China) in an ABI Prism 7500 Real-Time PCR System (Life tech, USA). The *TaActin* gene was used as an internal control for RT-qPCR. The relative expression levels of the target genes were calculated using the comparative 2^−ΔΔCT^ method (Livak and Schmittgen, 2001).

### BSMV-induced gene silencing in wheat CI12633

Barley yellow dwarf virus-mediated VIGS has been successfully utilized to study gene function in barley and wheat. In this study, a 207 bp fragment of *TaPSKR1L-6A* was subcloned into the *Nhe* I site of the BSMV RNA γ cDNA, forming the BSMV: *TaPSKR1L-6A* recombinant constructs. BSMV carrying empty GFP (BSMV: GFP) was used as control treatment. Then, these viruses were individually inoculated according to previous described methods (Zhu et al., 2017). After 15 days inoculated with BSMV, gene silencing efficiency was tested by RT-qPCR, and the silencing plants were inoculated with *R. cerealis* for disease resistance evaluation.

### Generation and identification of transgenic wheat

The full ORF sequence of *TaPSKR1L-6A* was amplified from cDNA of CI12633 plants. The full ORF sequence was added with a 6-His epitope tag and then was sub-cloned into monocot transformation vector pWMB110. The resulting pTaPSKR1L-6A-6*His vector, the transcript of the TaPSKR1L-6A-6*His was driven by the maize *ubiquitin* (Ubi) promoter and terminated by the 3’-non-transcribed region of the *Agrobacterium tumefaciens nopalinesynthase* (Tnos) gene (Wang et al., 2017). Subsequently, the pTaPSKR1L-6A-6*His vector was introduced into immature embryos of the wheat cv. ‘Yangmai 18’ via *Agrobacterium* mediated transformation. The transgenic wheat plants were detected via PCR with primers TaPSKR1L-6A-TF and Poly-R (Qi et al., 2024).

### *R. cerealis* inoculation and wheat sharp eyespot evaluation

The sharp eyespot inoculated and sharp eyespot evaluation methods were referred to the existing work of research (Chen et al., 2008; Qi et al., 2021).

The disease index calculation formula is as follows:


$$\text{DI}=\left\{\left(0\times \;{\text{X}}_{0}+1\times \;{\text{X}}_{1}\;+2\;\times {\text{X}}_{2}\;+\;3\;\times {\text{X}}_{3}\;+\;4\;\times \;{\text{X}}_{4}\;+\;5\;\times \;{\text{X}}_{5})\;÷[X_{0}\;+\;{\text{X}}_{1}\;+\;{\text{X}}_{2}\;+\;{\text{X}}_{3}\;+\;{\text{X}}_{4}\;+\;{\text{X}}_{5})\;\times 5\right]\right\}\;\times 100$$

The numbers 1–5 in the above formula represent the sharp eyespot infection types of different wheat plants, and X0-X9 represent the number of wheat plants in different disease infection types.

### Lignin staining, lignin content and stem mechanical (breaking) strength measurement

At 4 dpi with *R. cerealis*, basal leaf sheaths of T1 transgenic or WT ‘Yangmai 18’ plants were collected and stained with lignin staining. Lignin content was quantitatively measured by using the lignin ELISA Kit (ChemFaces, China) according to the manufacturer’s protocols. For lignin staining, fresh hand-cut sections of leaf sheaths were prepared from basal stems of wheat plants. Wiesner and Mäule staining methods were performed as previously described (Wang et al., 2018) and observed under an optical microscope. At harvest stage, the stem mechanical (breaking) strength of the second basal internode in *TaPSKR1L-6A* overexpressing and ‘Yangmai18’ wheat plants were measured using a Texture Analyser (TA) with a three-point bend test setup according a previous study (Miller et al., 2016).

## Data Availability

Due to agreements with the project funding agency, we are currently not permitted to provide the transcriptome data. Should you require access to transcript data of relevant genes from this study, please feel free to contact the corresponding author.

## Supplementary material

Supplementary Figure 1
*TaPSKR1L-6A* haplotypes are associated with sharp eyespot resistance. **(A)** Haplotypes of *TaPSKR1L-6A*. The SNP polymorphism at -1191 sit is shown in red. **(B)** Disease indexes of *TaPSKR1L-6A* haplotypes among 61 wheat cultivars. **(C)** Distribution of *TaPSKR1L-6A Hap I* and *Hap II* haplotypes in wheat cultivars.

Supplementary Figure 2 Stem mechanical (breaking) strength of the second basal internode in *TaPSKR1L-6A* overexpressing and ‘Yangmai18’ wheat plants at harvest stage.

Supplementary Table 1 Infection types and disease indexes of sharp eyespot in *TaPSKR1L-6A* -overexpressing and WT (‘Yangmai18’) wheat plants in T2 generation. Student’s *t*-test (***P* < 0.01).

Supplementary Table 2 Primers used in this study.
