# Supplementary material for: Overexpression of TaPSKR1L-6A improves resistance to sharp eyespot and increases lignin accumulation in wheat
Source: Front Plant Sci. 2025 Oct 17;16:1653282. doi: 10.3389/fpls.2025.1653282 (PMC12575328; doi:10.3389/fpls.2025.1653282)
Supplement: Supplementary Figure 1 — TaPSKR1L-6A haplotypes are associated with sharp eyespot resistance. (A) Haplotypes of TaPSKR1L-6A. The SNP polymorphism at -1191 sit is shown in red. (B) Disease indexes of TaPSKR1L-6A haplotypes among 61 wheat cultivars. (C) Distribution of TaPSKR1L-6A Hap I and Hap II haplotypes in wheat cultivars. [file DataSheet1.docx]

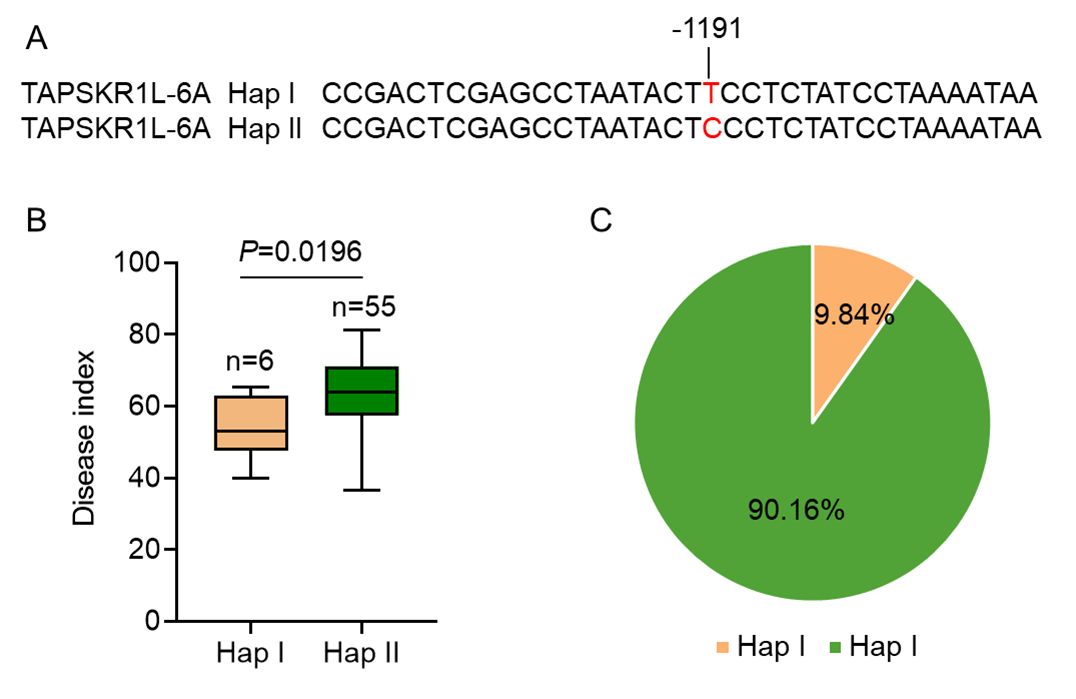


**Figure S1** *TaPSKR1L-6A* haplotypes are associated with sharp eyespot resistance. (A) Haplotypes of *TaPSKR1L-6A*. The SNP polymorphism at -1191 sit is shown in red. (B) Disease indexes of *TaPSKR1L-6A* haplotypes among 61 wheat cultivars. (C) Distribution of *TaPSKR1L-6A Hap I* and *Hap II* haplotypes in wheat cultivars.


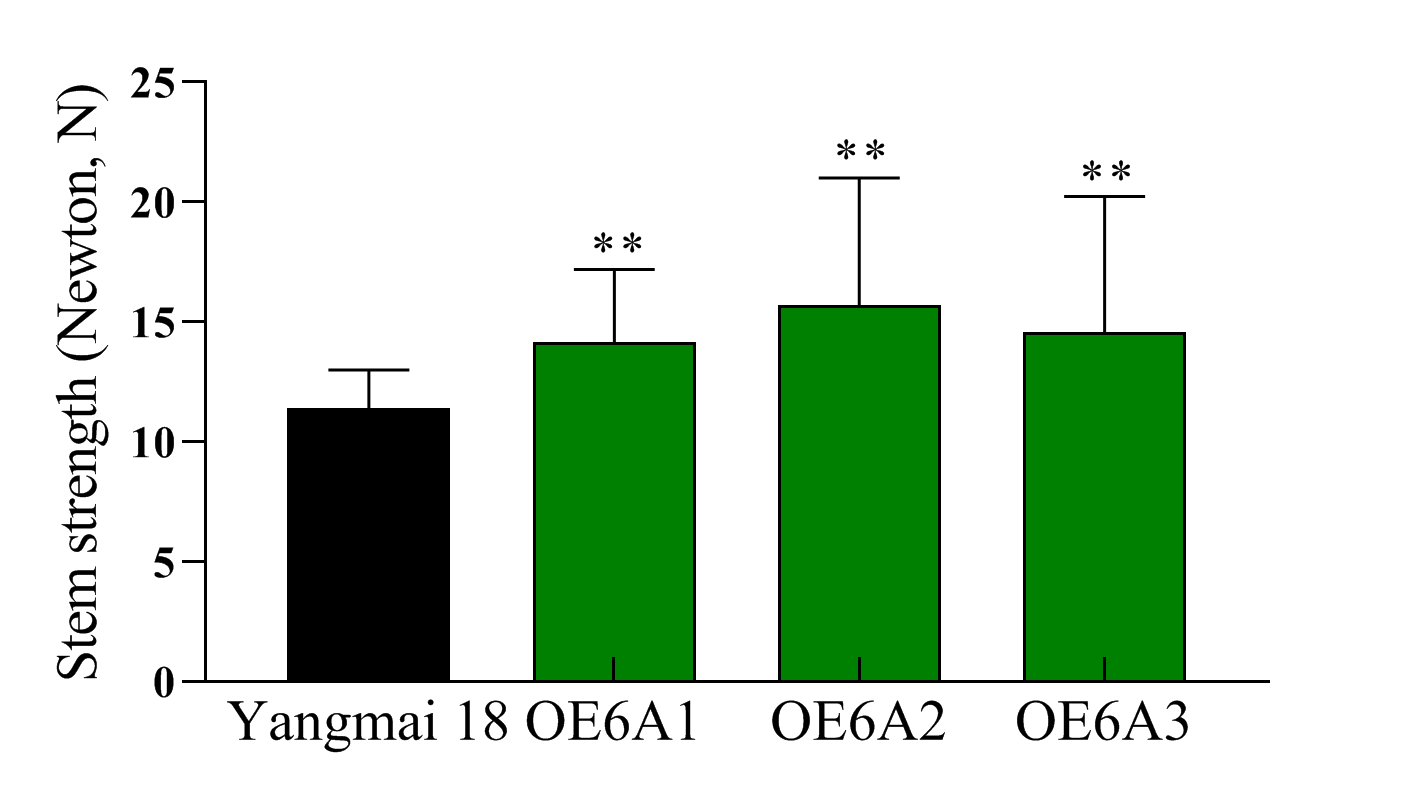


**Figure S2** Stem mechanical (breaking) strength of the second basal internode in *TaPSKR1L-6A* overexpressing and ‘Yangmai18’ wheat plants at harvest stage.
