# Supplementary material for: Overexpression of TaPSKR1L-6A improves resistance to sharp eyespot and increases lignin accumulation in wheat
Source: Front Plant Sci. 2025 Oct 17;16:1653282. doi: 10.3389/fpls.2025.1653282 (PMC12575328; doi:10.3389/fpls.2025.1653282)
Supplement: Supplementary Table 1 — Infection types and disease indexes of sharp eyespot in TaPSKR1L-6A -overexpressing and WT (‘Yangmai18’) wheat plants in T2 generation. Student’s t-test (**P < 0.01). [file Table1.docx]

**Table S1** Infection types and disease indexes of sharp eyespot in *TaPSKR1L-6A* -overexpressing and WT (‘Yangmai18’) wheat plants in T2 generation

| Lines | Number of wheat seedlings | Infection Types | Disease Index |
| --- | --- | --- | --- |
| WT (‘Yangmai 18’) | 39 | 2.60 | 52.16 |
| OE6A1 | 37 | 1.34** | 26.85** |
| OE6A2 | 35 | 1.19** | 23.93** |
| OE6A3 | 37 | 1.16** | 23.25** |

Student’s *t*-test (***P*<0.01).

**Table S2** Primers used in this study.

| Primer name | Sequence | Useage |
| --- | --- | --- |
| *VIGS-TaPSKR1LF* | 5'-TACGCTAGCGCCACTTTCAAAGGTGATG-3' | Primer for BSMV -VIGS assays |
| *VIGS-TaPSKR1LR* | 5'-GACGCTAGCAATCATCTTCATCATTTGC-3' |  |
| *VIGS-TaSERK1F* | 5'-TACGCTAGCACAACCTCCGGGCGGTGGAGC-3' |  |
| *VIGS-TaSERK1R* | 5'-GACGCTAGCCTGCAACTGGGAACTGCTGC-3' |  |
| *BSMV-CPF* | 5'-TGACTGCTAAGGGTGGAGGA-3' | Primer for CP gene |
| *BSMV-CPR* | 5'-CGGTTGAACATCACGAAGAGT-3' |  |
| *TaActin-RTF* | 5'-CGTCCATGATCCCTCCGAATT-3' | Primer for RT-qPCR |
| *TaActin-RTR* | 5'-CTCCATGTCATCCCAGTTG-3' |  |
| *TaPSKR1-RTF* | 5'-TGGCACACTGGGTTACATCC-3' |  |
| *TaPSKR1-RTR* | 5'-GCTCCCTTCCTCTTGCACAT-3' |  |
| *TaSERK1-RTF* | 5'-TGGCAGTGCATAGGAACCTG-3' |  |
| *TaSERK1-RTR* | 5'-CCCAGTGCAATCCGAGTTCT-3' |  |
| *TaCOMT3D-RTF* | 5'-CATCTACGCCAACGCATT-3' |  |
| *TaCOMT3D-RTR* | 5'-GAGGAAACACCAAGCCAAAG-3' |  |
| *TaPAL5-RTF* | 5'-ATGCGTGTGCGGTTCTCGTG-3' |  |
| *TaPAL5-RTR* | 5'-GATGTGCTTGCCTTGGTTCA-3' |  |
| *TaCAD1-RTF* | 5'-GGAGACGCAGGAGATGATGG-3' |  |
| *TaCAD1-RTR* | 5'-TCTGGTGACTAGAGCCTGGA-3' |  |
| *TaCCR1-RTF* | 5'-CGCCAAGAAGTACGCCAAC-3' |  |
| *TaCCR1-RTR* | 5'-CTTGTTGGGACGGGGTACTC-3' |  |
| *PWMB110-TaPSKR1F* | 5'-CGACTCTAGAGGATCCATGCGACGCACTACCACATGG-3' | Primer for  overexpression  vector construction |
| *PWMB110-TaPSKR1R* | 5'-ATCGGGGAAATTCGAGCTCTCAATGGTGATGGTGAT  GATGCTTTGTTGCTTCACTGCTAGC-3' |  |
| *TaPSKR1-RTF* | 5'-TGGCACACTGGGTTACATCC-3' | Primer for  transgenetic lines test |
| *Tnos-110-R* | 5'-CAAGACCGGCAACAGGATT-3' |  |
